# “He’s Still There”: How Facebook Facilitates Continuing Bonds With the Deceased

**DOI:** 10.1177/00302228211048672

**Published:** 2021-10-14

**Authors:** Charlotte Akinyemi, Alex Hassett

**Affiliations:** Salomons Institute, Canterbury Christ Church University, Tunbridge Wells, UK

**Keywords:** continuing bonds, bereavement, grief, Facebook

## Abstract

This study explored the processes involved when the bereaved use Facebook to continue bonds with the deceased. Grounded theory was used to analyze Facebook pages and interviews with bereaved Facebook users. Individual attempts at connection, such as posting about the deceased person, were bolstered by others witnessing and replying to the posts. Collective reminiscence occurred through the sharing of memories about the deceased, which sometimes led to learning new things about them. These individual and collective processes helped to maintain and transform a connection with the deceased person, who for some participants was “still there” on Facebook.

Klass et al. (1996) first introduced the term “continuing bonds” into bereavement literature, referring to a bereaved person maintaining an ongoing connection with the deceased as a common and normal part of grieving. Although the concept has existed throughout history across most cultures (Walter, 2018), it countered the dominant Western theories on bereavement during the 20th century that maintaining ties with the deceased indicated pathology in grief (Klass, 2006; Klass et al., 1996). Since the introduction of the term, it has been accepted by most psychological models of grief (Klass & Steffen, 2018, p. 4). Researchers have described a diverse range of ways in which continuing bonds can be expressed, including reminiscing about the deceased, telling stories about them, visiting their grave, looking at photographs or keeping their possessions (Root & Exline, 2014). Silverman and Nickman (1996) outlined five strategies of connection. These were locating the deceased, for example in “heaven”, experiencing the deceased, reaching out to the deceased, waking memories, and linking objects. The deceased may appear in dreams or nightmares, illusory or hallucinatory experiences, or through a sense of presence (Field et al., 2005).

Over the past two decades, continuing bonds have been revolutionized by the internet, which has supported and changed the processes of mourning in Western societies (Huberman, 2017). Previously physical processes are now largely digital, such as announcements of the death, attending funerals virtually, accessing grief support, and the public expression of grief. Earlier research into online mourning focused on memorial sites, also referred to as virtual cemeteries, cybermemorials or web memorials, which had been around since the late 1990s (Roberts & Vidal, 2000). The bereaved visited these sites more frequently than physical cemeteries (Roberts, 2004) and were more likely to access a memorial group than read a printed obituary (Carroll & Landry, 2010). The sites provided both a commemorative function and a means for the bereaved to communicate with the deceased (Huberman, 2017).

The latest platforms being used for this are social networking sites. By 2019, there were almost 3.5 billion social networking users worldwide (Chaffey, 2019), and in the UK, 66% of adults had used a social networking site in the past three months (Office for National Statistics, 2017). There is a wide variety of sites available, with some designed specifically to provide grief support. However, this research will focus on those most commonly used in daily life, such as Facebook. These sites which were previously used to socialize with the person are later used to mourn them. If the deceased person had a profile, mourning could also involve the person’s “representation of self” that they had created during their life (Kasket, 2012, p. 63). The technological development of smartphones has allowed these representations of the deceased to be accessible at any time (Walter, 2015).

## Facebook 

Since it opened to the general public in 2006, Facebook became of interest to researchers because of its potential functionality around death and bereavement. Facebook has 1.6 billion users who visit the site daily, and 2.5 billion monthly users (Facebook, 2020). When users pass away, relatives or friends inform Facebook of the death and their account can be “memorialized” (Facebook, 2018). Once memorialized, existing “friends” of the profile can view it, post on the wall and comment on one another’s posts. The bereaved have used Facebook to express grief related emotions and post memories of the deceased (Balk & Varga, 2018; Getty et al., 2011; Moyer & Enck, 2020), create community and maintain a continuing bond with the deceased (Rossetto et al., 2015).

## Maintaining a Continuing Bond Through Facebook

Qualitative studies have reported themes of the bereaved continuing a bond with the deceased though Facebook. This “preservation” of a connection occurred through learning more about the deceased, ongoing communication with them, and viewing posts that the deceased person had written before their death (Rossetto et al., 2015). Kasket (2012) stated that “the persisting digital self and the mourner's bond with it is experienced as somehow ‘real’, and there is a terrible fear of that bond being broken” (p. 63). Brubaker et al. (2013) named this continuation of the deceased a “post-mortem identity”. Several researchers have documented the phenomenon of the bereaved speaking directly to the deceased by posting on their profile page (Balk & Varga, 2018; Brubaker et al., 2013; Hieftje, 2012; Kasket, 2012). DeGroot (2012) coined the term “transcorporeal communication” to describe messages directed at the deceased as if they could read them. A “trigger” would prompt the person to communicate by sending a message to a representation of the deceased. This was either an inner representation or an external representation, such as their Facebook profile. They would receive feedback through what they thought that the deceased would say in response (DeGroot, 2018). A content analysis of profile pages of the deceased found that these types of messages increased in frequency over time since the loss (Bouc et al., 2016).

## Facebook Communities

Another dominant theme within the literature was the communal nature of grieving on Facebook. Hieftje (2012) stated that for the majority of the participants, Facebook “created a sense of community and belonging during their grief” (p. 41). Friends and family posting messages onto the deceased person’s profile publicly communicated their grief (Getty et al., 2011). Those who set up in-memory-of pages - a Facebook “page” dedicated to the memory of someone which others could “follow” - spoke about the support and information sharing that they were used for, and also how much they had learnt about the deceased person through the sharing of memories and photos on the pages (Kasket, 2012). Facebook facilitated connections with other mourners and functioned as a space for “sending, seeking and gaining” social support (Rossetto et al., 2015, p. 985). However, it could be painful to read others’ posts (Rossetto et al., 2015) and this community was not always found to be supportive, with instances of competition and conflict (Kasket, 2012). Privacy was a consideration and sometimes a challenge, where decisions had to be made about self-disclosure (Rossetto et al., 2015).

## Research Aims

The current literature indicates that continuing bonds with the deceased do occur through the use of Facebook, but there has been no model suggesting how this happens. For professionals working with bereaved individuals, “awareness of this fast-evolving phenomenon, and a framework for understanding it, are critical to providing effective bereavement support in the digital age” (Kasket, 2012, p. 9). This research therefore aims to answer the research question: how are bonds with the deceased continued through the use of Facebook?

## Materials and Methods

### Design

A qualitative grounded theory methodology was used (Glaser & Strauss, 1967). This allowed for the analysis of a variety of data and the formation of a theory. The data were coded using the Glaserian framework (Urquhart, 2013) and focused on social processes (Willig, 2012). The study collected data from two different sources, Facebook “in-memory-of” pages and interviews with bereaved Facebook users.

Facebook was selected for the research over other social networking sites because of its suitability for use around bereavement. Individual Facebook profiles continue after death and the personal content can be “memorialized”. Publicly accessible “pages” are used by communities of bereaved individuals. In addition, prior research into continuing bonds on social networking sites has mostly focused on Facebook.

### Participants

In phase one, 103 posts (including 388 comments) from seven “in-memory-of” Facebook pages were analyzed. Demographic information was collected about the deceased person to whom the page was dedicated. Three of the deceased were female and four were male. All of the sample were British, of which five were White British, one was Black British, and one was British South Asian. The time since the death ranged between 2 to 10 years prior (M = 5.6, SD = 4.4). Age at death ranged from 15 to 82 years old (M = 34.7, SD = 21.7).

In phase two, a different sample of seven participants was interviewed who had been bereaved by a family member (parent, grandparent or child), partner, or friend. Six were female and one was male. The age of participants ranged from 21 to 56 years old (M = 34.4, SD = 9.9). Five of the participants were White British, one participant was White British and Irish, and one was Filipino, and they all resided in the UK. All of the participants used Facebook on their smart phone daily, between 1 and 5 times per day. The time since the death ranged between 2 to 9 years prior (M = 4.4, SD = 2.2). The age at death ranged from 1 week old to 79 years old (M = 44.3, SD = 23.3), and the cause of death included a range of physical health conditions, homicide and suicide.

### Data Collection Procedure

For phase one, data were collected through “data mining” on Facebook (British Psychological Society [BPS], 2017; Russell, 2013). Publicly available Facebook pages were found by searching within Facebook, using the search terms “in memory of”, “in loving memory of” and “rest in peace”. Pages were purposively sampled, ensuring a range of gender, age at time of death and cause of death. Seven pages were sampled before “theoretical sufficiency” (Dey, 1999) was reached, which was a depth of understanding that was sufficient to develop categories and relationships between them, rather than a point where no new categories emerged.

For phase two, participants for interviews were recruited through Facebook advertisements and snowball recruitment. Participants needed to have used Facebook around a bereavement but were not required to have involvement with in-memory-of pages. Those who expressed interest in taking part were emailed information sheets and consent forms. An interview schedule was used as a guide, which had been developed following analysis of the phase one data and was amended following each interview. Participants were asked how they had used Facebook since the loss of the deceased person, how their use of Facebook had changed, and how this had impacted their grieving and feelings about the deceased person.

Interviews were conducted over the phone, audio recorded, and lasted between 20 to 60 minutes. One interview was conducted over email, being the participant’s preference.

### Data Analysis

A grounded theory approach was used for the analysis of the Facebook pages and interview data (Glaser & Strauss, 1967). In line with grounded theory best practice, data analysis was concurrent with the data collection (Urquhart, 2013), and ideas formed from the analysis then guided the theoretical sampling. The first author conducted the analysis, and the second author provided supervision. The data from Facebook pages were copied and pasted into a document and anonymized, and the audio recordings of interviews were transcribed verbatim. Analysis involved firstly open coding, where the data were coded line-by-line, then selective coding where the open codes were organized, and core variables were defined. Through a process of constant comparison, the data were abstracted into theoretical codes. Finally, this was developed into a model through the use of diagrams and theoretical memos. Although the data were collected in linear phases, the analysis moved back and forth between them in order to develop a theory.

### Ethical Considerations

The study received ethical approval from the university ethics panel. The BPS research board's ethical guidelines for internet-mediated research (BPS, 2017) were followed. For the online data, confidentiality and privacy was ensured by focusing on themes and trends rather than presenting raw data that would be highly personal and identifiable.

### Quality Assurance and Reflexivity

The quality of the research was assured through several processes (Yardley, 2000). This included the first author partaking in a bracketing interview with a peer prior to data collection, to become aware of some of the biases and preconceptions held about the research. During the initial participant recruitment, a disconfirming case (Yardley, 2008) was sought through theoretical sampling and formed the basis of the “disengaged” theme. During analysis, the first author used coding memos to document the process of coding and development of categories and theory. Regular discussion with the second author, acting as research supervisor, allowed for reviews of the coding, categories and developing theory.

## Results

**Figure 1.**
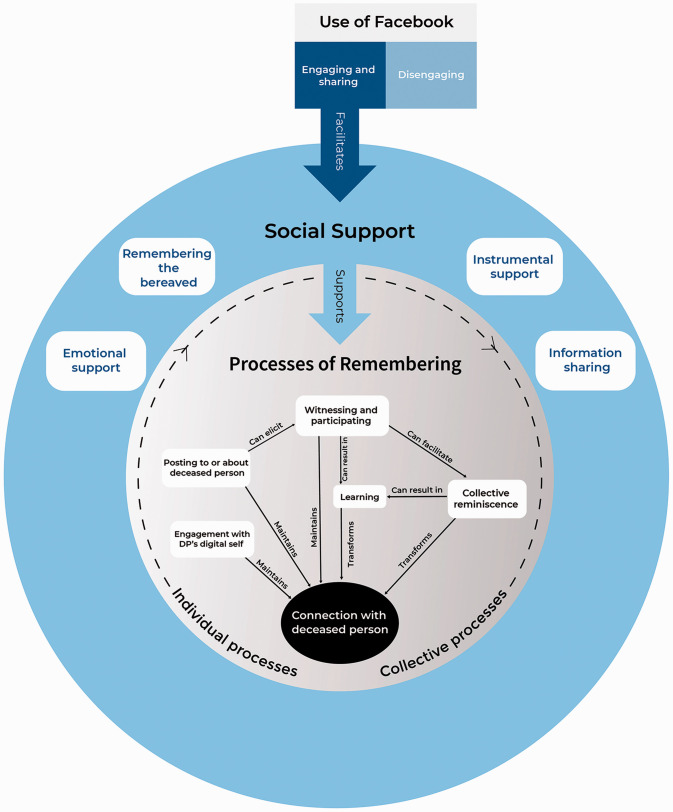
Continuing Bonds on Facebook Through Individual and Collective Remembering.

Four categories emerged, describing how bonds with the deceased were continued through Facebook. These were: Facebook use, social support, processes of remembering and connection with the deceased. Figure 1 illustrates the interactions between these categories. This theory relates to those who engage with Facebook in relation to their bereavement and are involved in sharing thoughts, feelings and memories through Facebook. The theory suggests that the bereaved and their community on Facebook remember the deceased person through various individual and collective processes. The bereaved are remembered and supported by a Facebook community. This in turn supports remembering of the deceased person, which includes engaging with the deceased’s Facebook profile and posting to and about the deceased person. These individual attempts at connection are bolstered by others witnessing and responding to posts, collectively reminiscing about the deceased person and learning new things about them from each other’s memories. These processes of remembering help to maintain and transform a connection with the deceased person, which can involve a feeling that the deceased person is “still there” on Facebook.

### Category 1: Facebook Use

The first category portrayed how participants used Facebook. Every participant had made decisions about engagement with Facebook. They described or inferred occasions when they would engage or disengage. When they engaged, they sought information through Facebook, shared and responded to others. Times when they disengaged, they avoided Facebook or certain aspects of it.

#### Subcategory 1: Engaging and Sharing

Five participants spoke about making decisions regarding using Facebook, including how much to share, what to share, when to respond and what to follow. Participant 2 said “I struggle with posting pictures of [deceased person], so just have a select few I choose that have been edited, softened, or are black and white”. For participant 1, after his grandmother died, he said “I was hardly posting anything, and then when I first saw my cousin post something about it, I was like ah maybe I should post something as well”. Participant 5 thought this was an advantage of Facebook compared to face-to-face conversations, as it was less intrusive and you could make choices about responding.

#### Subcategory 2: Disengaging

Three participants spoke about disengaging from Facebook. Participant 6 was sought through a process of theoretical sampling, due to her negative experiences of using Facebook. She spoke about finding it too difficult to engage with Facebook around her friend’s death. She felt harassed by friends of the deceased person. She also avoided information about the death, and later reminders of it. She said that her friend had a Facebook profile but that “I ended up deleting her on Facebook because I didn’t want to know she passed”. She avoided the posts of Facebook grief groups on her newsfeed, because it would remind her of her loss, and sometimes “unfollowed” the group. Participant 5 said she found a grief group helpful initially, but later related to it less. Although she continued to follow the group, she no longer responded to the posts. Participant 7 spoke about disengaging from Facebook for several weeks following her father’s death because she would use Facebook less when she felt in a low mood.

### Category 2: Social Support

Social support was seen to occur on the in-memory-of pages. Those whom the person was in contact with through Facebook are here referred to as the “community”. This included family, friends, and a wider group of people who did not know the deceased person, such as support groups. As Facebook was used internationally, this support and information sharing occurred across geographical boundaries. The support was typically being sought and received by the bereaved participants, but there were instances where participants provided support to bereaved others.

#### Subcategory 1: Remembering the Bereaved

In phase one of the data collection, condolences and messages were sent by members of the community to the directly bereaved on the in-memory-of pages. Phrases such as “thinking of you” indicated that the community were remembering the bereaved person and their grief. In phase two, five participants spoke about it being important that they and their grief were not forgotten by the community on Facebook. Participant 3 described it as “that sense of unity that actually they haven’t forgotten us, they haven’t forgotten that we’re still grieving every single day”. Receiving emotional support can also contribute to the feeling of being remembered by the community.

#### Subcategory 2: Emotional Support

On the in-memory-of pages, emotional support was seen through the expression of emotions in messages, such as shock and sadness, which were shared by the bereaved and the community. The community would send their love to the bereaved, through words and sometimes emojis. In phase two, four participants also spoke about receiving emotional support from their Facebook community, through others expressing understanding and caring. For participant 4, it was her existing Facebook friends who reached out after she posted about her late father. She said, “it’s been really nice to have other people in my friends list, that I didn’t even know had been through a similar thing, reach out”.

#### Subcategory 3: Instrumental Support

Some social support came in the form of instrumental, tangible support, both on and offline. This was seen on the in-memory-of pages and mentioned in three of the interviews. For participant 5, the loss of her husband had left her with a house to maintain alone and posting on Facebook resulted in help from friends of her late husband. She said, “I can just post a comment, oh fed up with that, or this has happened, that’s a bad start to the day, and someone will go don’t worry I’ll sort it for you”. Another common use of Facebook following a bereavement was to fundraise for charities related to the person’s death by posting links to online fundraising pages along with updates on the progress of such projects.

#### Subcategory 4: Information Sharing

Bereaved people were seen to share information with others on the in-memory-of pages about the death and funeral arrangements. Interviewees had commonly used Facebook in this way following a death. Participant 5 used the deceased person’s Facebook account to contact the deceased’s friends and colleagues and let them know that he had passed. Participant 2 sought information by following national organizations and charities on Facebook that were related to the death of her son. She described how this “brought me awareness of baby loss wave of light – lighting a candle […] every year with #waveoflight. I like that they use facts and research to try and gain awareness in prevention of premature birth”.

### Category 3: Processes of Remembering

The deceased person was remembered through various processes. Some of these were individual processes, such as posting on the deceased’s profile to wish them a happy birthday. At other times the remembering was through collective processes. This involved more than one person posting about the deceased then witnessing and responding to one another’s posts, often through comments. The dashed line in Figure 1 represents the flow of the individual processes eliciting collective processes.

#### Subcategory 1: Engagement With Deceased’s Digital Self

Four participants continued to engage with the deceased person’s profile following their death, sometimes for many years. Participant 5 described looking back over things that had been posted by the person before their death and how that aided remembering. She said, “you can see video, you can see normal interactions, you can look back over posts and think I remember when he said that stupid thing and yeah it just keeps it all fresh”. Participant 3 preserved the page as it had been when the person was alive and found this comforting.

#### Subcategory 2: Posting to or About Deceased Person

The deceased was remembered by the bereaved and their Facebook community, through a process of posting and commenting to or about the deceased person. Five of the participants mentioned this process, and it was seen extensively across the in-memory-of pages. One example was the expression of missing or love for the deceased person. Participant 7 described this as “the way of [saying] I miss you Dad, I love you Dad, erm and instead of just sort of saying it or thinking it, you share it on Facebook”. Posts can also include descriptions of deceased’s character or sharing photos and memories of them. Posts can even be written in a way that is directed to the deceased person, for example posting on the deceased person’s wall, tagging them in posts, wishing them a happy birthday, or marking other anniversaries or holidays.

#### Subcategory 3: Witnessing and Participating

The posts analyzed from the in-memory-of pages often had comments below, which exhibited how others had witnessed and participated in the remembering of the deceased. The bereaved person would post photographs of the deceased person, and others would comment on them with their thoughts, feelings and memories in relation to the photographs. Four participants also spoke about witnessing others post about the deceased person. Participant 3 said, “I love seeing people’s photos of him. I love seeing people erm sharing memories or putting something up. I mean sometimes people will share photos from 20, 30 years ago”. Some participants were consciously aware that they would post to remind the community to remember the deceased person. Participant 7 said, “because it’s on Facebook […] it comes up doesn’t it, so and so’s posted this, friends might see, and they’ll think of him perhaps”. For participant 2, it was a direct way to make sure others did not forget about the deceased, saying, “I like when people comment or like my post as I feel it helps me to remind people not forget about him”.

#### Subcategory 4: Collective Reminiscence

Participant 5 described a “little moment of reminiscence” happening collectively on Facebook between the bereaved and the community. It occurred through a mutual sharing of memories of the deceased person, through posting about the deceased person and others witnessing and commenting on those posts.

Then everybody else puts their memories and thoughts in as well and you just get a little moment of reminiscence, as if you were all collected together at a social event or something […] you know someone will have something and go ‘oh I remember when this one with [deceased person]’ and lots of other people will jump in and go ‘oh yeah’.

Participant 3 referred to a “shared sense of memory”, that had been present immediately after the death, but occurred less frequently as others moved on with their lives. She described it as “a way of just having that shared feeling again with other people, his friends, with extended family, with people that we might see”. This process could also be seen within the in-memory-of pages, such as someone posting a photo of the deceased on a motorbike and asking if anyone else remembered how much he loved his motorbike. Others responded by leaving comments under the post of their own related memories.

#### Subcategory 5: Learning

Three participants described learning new things about the deceased person. This could occur through witnessing others posts to or about the deceased person or through participating in collective reminiscence. Sometimes, as a result of others sharing such memories, the person would garner new knowledge about the deceased. Participant 5 learnt more about her late husband from his colleagues by witnessing memories of him in their posts.

I got to hear about a side of him that I didn’t really know. Because he wasn’t one for making a big deal of himself, but he’d clearly made a huge impression on a lot of people because he was very supportive and encouraging.

After his grandmother died, participant 1 found out more about her life, and said this was directly linked to a feeling of his grandmother “continuing to live with us”.

### Category 4: Connection with Deceased Person

The outcome of the above processes of remembering, was that a connection with the deceased person was maintained and even transformed. Within the in-memory-of pages, there were many mentions that the deceased were still loved, still in the thoughts of the bereaved, and would never be forgotten. Words such as “eternity”, “forever”, and “always” were frequently used in the posts and comments to express this ongoing connection. In phase two, there was a belief described by three of the participants about the deceased person figuratively continuing to live through Facebook. Remarks from the participants were that the deceased was “still with us” (P1), “he’s still there” (P7) and “it keeps him alive” (P5). They all clarified that they did not mean this literally, but that figuratively the deceased were thought to be living on through Facebook.

Participant 7 explained this in context of her father’s Facebook profile remaining on her list of Facebook friends, and that he was still part of that shared space. She also spoke about writing posts in a way that was directed at her father, as a way of “contacting him”. For participant 3, she had a similar experience and also noticed others writing directly to her stepfather.

When I’ve seen other people’s messages as well, they’re all as if [deceased person’s name] will be reading them, even though we know that he isn’t. You know, we’re not, we understand, but it is a nice feature of Facebook because then people might comment, or the family might like the post. It’s just another way to keep his memory alive.

## Discussion

### Processes of Remembering

This research provides a model for understanding how bonds with the deceased are continued through Facebook. The “processes of remembering” part of the model outlines how individual attempts at remembering the deceased through Facebook can become collective due to the design of the site. Posts are inevitably witnessed by others, who participate in posting and responding. Memories of the deceased are shared and discussed, facilitating collective reminiscence. The individual learns more about the deceased (Kasket, 2012), and their connection to the deceased is not only maintained, as was reported in previous research (Rossetto et al., 2015), but also transformed through the input of others into these collective remembering processes. The centrality of collective processes in this model supports researchers who have recommended that understanding communal continuing bonds, and their intersubjectivity, is an area in need of development (Klass, 2006; Klass & Steffen, 2018; Walter, 1996). Hartman (2012) aptly described the process: “cyberspace is transforming loss into a collective event endowing the lost object with a new kind of immortality” (p. 455).

For the individual processes of remembering, the themes reported in the present study fit within the existing literature. Posting to the deceased person could be described using DeGroot’s (2012) term “transcorporeal communication”, which had also been related to keeping the deceased “alive”. Engagement with the deceased’s digital self was already a well-documented phenomenon and had been referred to by various terms across the literature (Brubaker et al., 2013; Kasket, 2012; Rossetto et al., 2015).

### Connection With Deceased Person

The research conceptualizes a connection with the deceased person as the outcome of the processes of remembering. This fitted with continuing bonds theory that predated social networking sites, where the purpose of grief is “the construction of a durable biography that enables the living to integrate the memory of the dead into their ongoing lives; the process by which this is achieved is principally conversations with others” (Walter, 1996, p. 7). These conversations are seen in the witnessing, participating, and collective reminiscence processes.

The model does not include aspects around whether the connection was helpful or unhelpful to the bereaved person, or how it may have impacted or been influenced by their grieving. This is because experiences varied considerably between the participants. Some who engaged with Facebook found it comforting to have that connection and all that came with it. This included the social support, the profile page to visit, and the collective processes of remembering in which to participate. However, other participants found elements, or all of it, distressing. Seeing content related to the deceased or other people’s bereavements led them to disengage from the particular page or Facebook altogether. For some it was uncomfortable to see the decline in the participation of others. One participant said that she was the only person who continued to leave messages on the deceased’s profile. However, as the helpfulness was not the focus of the research, conclusions were not made about this aspect. Further research could explore this in more depth (Blower & Sharman, 2021).

### Social Support

At the social support level of the model, the experiences of participants being remembered and supported by others through Facebook, reflected the findings of previous research (Hieftje, 2012; Rossetto et al., 2015). The social support of the Facebook communities fitted within the different types of social support theorized more broadly, where supportive behaviors were categorized into instrumental, informational, emotional and appraisal (House, 1981). However, appraisal support was not likely to be highlighted in phase two as participants did not usually report the language or words used by others to support them. The support was also bidirectional (Li et al., 2015). Participants spoke of receiving the support, but also mentioned offering support to others.

However, even social support was not always experienced as helpful. It was perceived by some participants as overwhelming and unwanted, including those who disengaged from Facebook grief groups or had felt harassed by the floods of messages following a death. Social support as a negative experience has been described in other research, particularly in relation to online support groups (Palant & Himmel, 2019).

### Limitations

This study had several limitations. To separate out Facebook use from other social media could lack ecological validity. In daily life people may use multiple different platforms and switch between them seamlessly on their smartphone. Most of the participants mentioned other sites which they also used, such as Instagram and Reddit, and messaging applications such as WhatsApp.

Another limitation was that participants’ religion was not collected as part of the demographics. There has been a tension in the literature between viewing Facebook as a generally secular space, and the importance of pre-existing religious, cultural or spiritual beliefs. The continuing bonds literature has been criticized in general for its lack of consideration of religious and spiritual beliefs (Root & Exline, 2014).

Participants for phase two were recruited through responding to a Facebook advertisement and were therefore self-selected. These were people for whom the research had a particular resonance and so were unlikely to represent a wide spectrum of Facebook users who experience bereavement. This limits the generalizability of the research. The model attempts to account for this with the “Facebook use” category, which includes a theme for those who disengage from Facebook or avoid using it around a bereavement. Other limitations are the small sample and lack of ethnic diversity as the majority of participants were White British.

### Implications for Research

This research builds on the continuing bonds theory, and 14 years of bereavement research into Facebook, by suggesting a model for understanding how bonds with the deceased are continued through Facebook. The dominance of collective processes in the model supports the call for more attention on the intersubjective and co-constructed elements of continuing bonds online (Klass & Steffen, 2018).

One of the participants in this study preferred to respond to an interview schedule over email. As other bereavement research had found that those who felt that they would be too distressed to talk would decline participation (Epstein et al., 2006), it may be useful for future research to incorporate such methods of data collection.

### Implications for Practice

The current research had initially planned to interview bereavement therapists. However, it was unsuccessful in recruiting any therapists who had experience of speaking with their clients about Facebook use. This could have been due to clinicians’ lack of awareness or confidence to address the subject. This research could encourage those conversations in therapy by providing a framework for clinicians to understand their clients’ engagement or disengagement from Facebook in relation to a bereavement. If clients are choosing to engage with Facebook, it may be helpful to assess their experience of this, and how it might maintain and transform a connection with the deceased.

Online interactions may be thought of as “second rate” by therapists (Hartman, 2012, p. 455), however this research demonstrates how integral and significant online interactions can be for the bereaved. As a result of the COVID-19 pandemic, there has been a migration of therapy onto online video calling platforms (BPS, 2020). Some may be concerned that this is too disconnected, but this research would suggest that it be viewed as an opportunity to appreciate and enhance our understanding of the online interactions that we now have with the living, the deceased, and the therapist.

This grounded theory has the potential of wider implications beyond Facebook. The model could be generalized to other sites that have sufficient features, such as Instagram and Twitter. These sites would need to allow for the social support and processes of remembering. For processes of remembering to occur sites must include a digital representation of the deceased, an ability to post to and about the deceased, and the option to comment on one another’s posts. Furthermore, although the concept of processes of remembering was generated in relation to online use, it may also be relevant in explaining how connections with the deceased occur offline through verbal collective remembering. Posting and witnessing could be replaced with talking and listening. Participating could refer to having a conversation about the deceased. In this case, they could similarly result in collective reminiscence and learning. Further research could explore the extent of the online to offline translation of the findings.
